# Improving the Prediction of Radiation Pneumonitis: Leveraging Radiomics and Dosiomics Within IDLSS Lung Subregions

**DOI:** 10.3390/life16020328

**Published:** 2026-02-13

**Authors:** Tsair-Fwu Lee, Wen-Ping Yun, Ling-Chuan Chang-Chien, Hung-Yu Chang, Yi-Lun Liao, Ya-Shin Kuan, Chiu-Feng Chiu, Cheng-Shie Wuu, Yang-Wei Hsieh, Liyun Chang, Yu-Chang Hu, Yu-Wei Lin, Pei-Ju Chao

**Affiliations:** 1 Medical Physics and Informatics Laboratory of Electronics Engineering, National Kaohsiung University of Science and Technology, Kaohsiung 80778, Taiwan; tflee@nkust.edu.tw (T.-F.L.); 1106405106@nkust.edu.tw (W.-P.Y.); wewe750422@gmail.com (Y.-W.H.); 2 Graduate Institute of Clinical Medicine, Kaohsiung Medical University, Kaohsiung 807378, Taiwan; 3 Department of Medical Imaging and Radiological Sciences, Kaohsiung Medical University, Kaohsiung 80708, Taiwan; 4 CROSS GRACE, Dental Clinic, Kaohsiung 804314, Taiwan; 5 Department of Radiation Oncology, Columbia University, New York, NY 10032, USA; csw6@columbia.edu; 6 Department of Radiation Oncology, Kaohsiung Veterans General Hospital, Kaohsiung 813414, Taiwan; 7 Department of Medical Imaging and Radiological Sciences, I-Shou University, Kaohsiung 82445, Taiwan; cliyun2000@gmail.com; 8 Department of Radiation Oncology, Kaohsiung Chang Gung Memorial Hospital, Chang Gung University College of Medicine, Kaohsiung 833253, Taiwan

**Keywords:** radiation pneumonia, volume-modulated arc therapy, radiomics, dosiomics, machine learning, incremental-dose interval-based lung subregion segmentation (IDLSS)

## Abstract

**Purpose:** This study develops a predictive model for radiation pneumonitis (RP) risk in lung cancer patients after volume-modulated arc therapy (VMAT) that leverages high-dimensional dosiomics and dose–volume histogram (DVH) features within IDLSS (incremental-dose interval-based lung subregion) lung subregions. **Methods:** We retrospectively analyzed data from 136 lung cancer patients treated with VMAT between 2015 and 2022, including 39 patients who developed RP greater than Grade 2. Using the IDLSS method, seven regions of interest (ROIs), including the Planning Target Volume (PTV), normal lung, and five subdivided lung areas, were delineated on pretreatment Computed Tomography (CT) images. DVH, radiomics, and dosiomics features were extracted from these ROIs and organized into nine distinct feature sets. A comprehensive pipeline was applied, integrating IDLSS-defined lung subregions, high-dimensional dosiomics features, LASSO-based feature selection, and SMOTE oversampling to address class imbalance in the training data. Logistic regression, random forest, and feedforward neural networks were constructed and optimized via tenfold cross-validation. Model performance across different feature sets was evaluated via the average AUC, F1 score, and other performance metrics. **Results:** LASSO regression revealed that BMI and volume within the 5–10 Gy and 10–20 Gy lung subregions were significant predictors of RP. The performance evaluation demonstrated that the dosiomics features consistently outperformed the DVH features across the models. Combining radiomics and dosiomics achieved the highest predictive accuracy (AUC = 0.91, ACC = 0.89, NPV = 0.95, PPV = 0.78, F1 score = 0.82, sensitivity = 0.88, specificity = 0.90). Applying SMOTE during training significantly improved sensitivity without compromising specificity, confirming the value of balancing strategies in enhancing model performance. Incorporating all the features together did not provide additional performance gains. **Conclusions:** Integrating radiomics and dosiomics features extracted from IDLSS-defined lung subregions significantly enhances the ability to predict RP after VMAT, surpassing traditional DVH metrics. The substantial contribution of dosiomics features highlights the importance of spatial dose heterogeneity in RP risk assessment.

## 1. Introduction

This study aims to increase the safety and efficacy of volume-modulated arc therapy (VMAT) [1], particularly in the prevention and prediction of radiation pneumonitis (RP) [2,3]. RP is a common and potentially severe complication following lung cancer radiotherapy that can significantly impair patients’ quality of life and survival rates [4,5]. Although VMAT technology improves beam precision and reduces damage to healthy tissues, the reported incidence of RP in lung cancer patients still ranges from 17% to 47% [6,7], highlighting the need for more accurate predictive tools to identify high-risk patients prior to treatment.

Traditional RP risk prediction relies primarily on dose–volume histogram (DVH) parameters, which summarize the relationship between radiation dose and lung volume [8]. However, conventional DVH-based approaches average the dose across the entire lung and neglect spatial dose heterogeneity, which may contain critical information about localized tissue responses that contribute to RP development. This spatial oversimplification reduces the ability to identify high-risk subregions within the lung.

To overcome these limitations, this study adopts a high-dimensional feature integration strategy that combines radiomic features derived from pretreatment CT images [9,10,11,12,13] with dosiomics features extracted directly from dose distributions [14,15]. Compared with DVH, dosiomics provides richer spatial-dose descriptors, reflecting spatial heterogeneity in dose delivery [16,17,18]. Radiomics, on the other hand, captures tumor and normal tissue texture, intensity, and shape characteristics, which may reflect tissue sensitivity and inflammatory responses linked to RP. Integrating these two complementary omics methods offers a more holistic and biologically informed approach to RP risk prediction.

A key innovation of this study is the introduction of the incremental-dose interval-based lung subregion segmentation (IDLSS) method, which was originally proposed by Bing Li et al. [19]. IDLSS refines conventional whole-lung feature extraction by dividing the lung into multiple dose-specific subregions. Specifically, IDLSS stratifies the lung into incremental dose intervals (e.g., 5–10 Gy, 10–20 Gy, 20–30 Gy, etc.), creating spatially stratified ROIs that preserve dose heterogeneity within clinically relevant dose ranges. This enables spatially aware feature extraction, linking both radiomic and dosiomic features directly to distinct dose subregions rather than averaging across the whole lung. By focusing on localized high-risk regions where biological damage is more likely to occur, IDLSS enhances the biological interpretability of the extracted features and improves predictive performance.

Furthermore, to address the inherent class imbalance between RP-positive and RP-negative cases, this study employs the synthetic minority oversampling technique (SMOTE), ensuring balanced model training and improving sensitivity to minority class (RP-positive) cases.

The overall objective of this study was to develop and evaluate machine learning models that integrate these multidimensional and high-dimensional features to predict RP risk in lung cancer patients undergoing VMAT. By systematically comparing the predictive performance of DVH, radiomics, and dosiomics features—and their combinations—across multiple models, this study aims to identify the optimal feature set and modeling approach to increase RP prediction accuracy.

Beyond conventional performance metrics, this study further employed decision curve analysis (DCA) to evaluate the clinical utility of each model by quantifying the net clinical benefit across different decision thresholds. DCA directly reflects the value of predictive models in real-world clinical decision-making and provides critical evidence for their practical applicability. In recent years, studies by Slimi et al. [20], such as those incorporating attention-guided spiking neural network–gated recurrent unit (SNN–GRU) hybrid architectures, have enhanced the reliability of clinical prediction by improving robustness to imaging noise and model interpretability. This line of research is highly aligned with the core objective of the present study, which focuses on validating the tangible clinical net benefit provided by predictive models through DCA.

This study pioneers a novel framework that places the incremental-dose interval-based lung subregion segmentation (IDLSS) method at its core, combining it with radiomics–dosiomics feature extraction, LASSO feature selection, and SMOTE-based class balancing to form a comprehensive and interpretable RP risk prediction pipeline. Unlike conventional whole-lung feature extraction methods, IDLSS preserves spatial dose heterogeneity and explicitly links each subregion’s features to biologically relevant dose intervals, ensuring that both imaging-derived radiomic signatures and dose-derived dosiomic patterns are spatially and biologically grounded. This spatially resolved strategy not only refines radiotherapy outcome prediction but also enhances clinical interpretability by aligning predictive features with localized dose regions that matter most in RP development.

Building upon this core, this study highlights a comprehensive and clinically oriented predictive framework that integrates IDLSS-guided spatial feature extraction, radiomics–dosiomics fusion, LASSO-based feature selection, and SMOTE oversampling into a single pipeline. This integrated approach not only enhances predictive accuracy but also offers interpretable, biologically meaningful, and clinically actionable insights for personalized radiotherapy planning. The findings are expected to inform future treatment strategies, reduce RP incidence, improve patient quality of life, and provide technical and clinical references for precision radiotherapy.

## 2. Key Contributions

The main contributions of this study can be summarized as follows:

- IDLSS-guided spatial feature extraction: This study places the incremental-dose interval-based lung subregion segmentation (IDLSS) method at the core of the framework, enabling high-dimensional radiomic and dosiomic feature extraction from dose-specific lung subregions (5–50 Gy) and preserving spatial dose heterogeneity that is overlooked by whole-lung analyses.
- Multimodal feature fusion: By integrating radiomics, dosiomics, clinical factors (e.g., BMI), and conventional DVH parameters within a unified modeling framework, this study leverages complementary cross-domain information to enhance RP risk prediction performance.
- Clinical utility validation via decision curve analysis (DCA): Beyond conventional performance metrics, decision curve analysis was employed to quantify net clinical benefit across different decision thresholds, demonstrating the practical value of the proposed framework for personalized radiotherapy decision-making.

## 3. Materials and Methods

### 3.1. Research Framework

**Figure 1:**
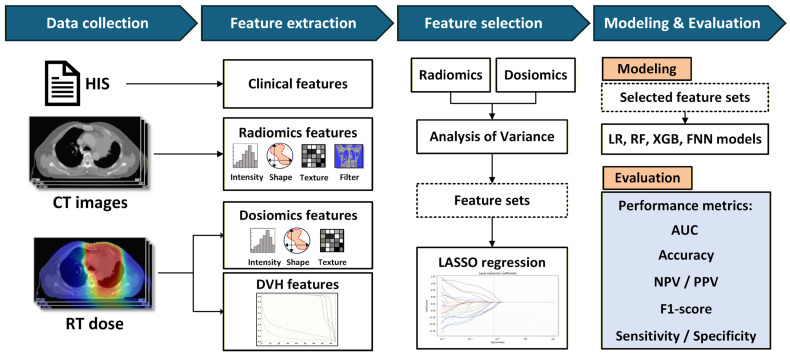
Overview of the Research Process, Including Data Collection, Feature Extraction, Feature Selection, Model Development, and Evaluation. Abbreviations: HIS: Hospital Information Systems, CT: Computed Tomography, RT: Radiation Therapy, DVH: Dose–Volume Histogram, LASSO: Least Absolute Shrinkage and Selection Operator, LR: Logistic regression, RF: Random Forest, FNN: Feedforward Neural Network, AUC: Area Under the ROC Curve, NPV: Negative Predictive Value, PPV: Positive Predictive Value. **Explanation of the Arrows:** The arrows represent the sequential data pipeline. They indicate the flow of information starting from multimodal data collection (HIS and CT images), through automated feature extraction and statistical selection (ANOVA and LASSO), into machine learning model construction, and finally to performance validation using clinical metrics. **Explanation of Colors (Workflow Stages):** Dark Blue Headers: Represent the four primary phases of the study: Data Collection, Feature Extraction, Feature Selection, and Modeling & Evaluation. Light Blue/White Boxes: Indicate the specific tools and datasets used, such as CT images, RT dose distributions, and performance metrics like AUC, Accuracy, and F1-score. Orange Highlights: Denote the core modeling and evaluation components, distinguishing the machine learning algorithms (LR, RF, FNN) from the preceding data processing steps.

This study aimed to establish a predictive model for assessing the risk of RP in lung cancer patients after VMAT. The overall process is illustrated in Figure 1. The workflow begins with data collection, including clinical data, pretreatment CT images, and dose information from radiotherapy plans. These data were used for feature extraction and selection, followed by model development and performance evaluation. The primary goal was to utilize multisource data and high-dimensional features to construct a robust predictive model, eventually enabling RP risk prediction after radiotherapy.

To examine the predictive performance of different types of features, we constructed models using various feature combinations, including DVH features (representing traditional two-dimensional dose–volume relationships) and dosiomics features (representing spatial dose heterogeneity through texture analysis). Initially, logistic regression (LR) was selected as the baseline model because of its interpretability, computational efficiency, and reliability in medical prediction tasks. To further evaluate the robustness of our findings, we also employed random forest (RF), Extreme Gradient Boosting (XGB) and feedforward neural network (FNN) classifiers to compare different machine learning approaches in handling high-dimensional radiomic and dosiomic data.

Finally, we systematically compared the performance of these models across various feature sets. The objective was to evaluate the alignment between specific feature types—particularly high-dimensional dosiomics—and different modeling techniques, thereby identifying the optimal strategy to enhance predictive accuracy for RP risk.

### 3.2. Data Collection

This study was a retrospective observation of 136 lung cancer patients who received VMAT treatment between 2015 and 2022, with prescribed doses greater than 50 Gy. Data collection included pretreatment CT images, treatment plan doses, and DICOM patient data. Age, sex, BMI, tumor TNM stage, and chemotherapy records were also recorded. This study involving human participants was approved by the institutional review board (IRB) at Kaohsiung Veterans General Hospital, with approval number KSVGH23-CT12-03. Standards and regulatory requirements were followed, and the need for informed consent was waived.

### 3.3. Radiation Pneumonitis Assessment

In this study, patient follow-up records after treatment were retrospectively analyzed to determine the occurrence of RP. RP reactions in lung cancer patients were assessed by professional radiation oncologists according to the criteria established by the RTOG on the basis of age and CT. The grading criteria for RP are as follows: Grade 0 indicates no apparent radiation-induced lung changes; Grade 1 represents mild radiation pneumonitis, characterized by a mild inflammatory response, with patients possibly experiencing slight cough refractory or daily activities; Grade 2 refers to moderate radiation pneumonitis, accompanied by significant symptoms such as persistent cough and difficulty breathing, which may require medical intervention and drug treatment; Grade 3 is severe radiation pneumonitis, with patients experiencing severe breathing difficulties, requiring oxygen therapy or more aggressive treatment measures; and Grade 4 is extremely severe radiation pneumonitis, which may lead to permanent lung damage or require life support equipment. In this study, RP predicted to be Grade 2 or above (≥Grade 2) was defined as a significant response. The clinical data and records of the 136 RP patients are shown in Table 1.

### 3.4. Feature Extraction

**Figure 2:**
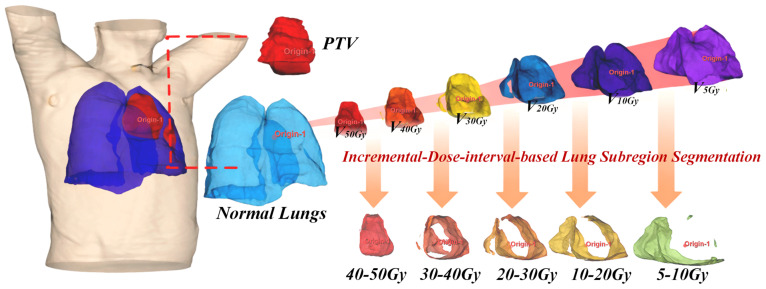
Schematic of the seven Regions of Interest (ROIs) used in this study, where IDLSS is derived by subtracting VxGy from each other. Abbreviations: PTV: Planning target volume, IDLSS: Incremental–dose interval–based lung subregion segmentation. **The Arrows:** The arrows represent the mathematical process of deriving specific subregions. IDLSS subregions are generated by subtracting different dose range curves (VxGy) of the normal lungs from each other to capture spatial dose heterogeneity. **Color Coding for Subregions:** The subregions are partitioned based on dose gradients ranging from 5 to 50 Gy: Red: Planning Target Volume (PTV) and the 40–50 Gy subregion. Yellow: Normal Lung (whole) and the 30–40 Gy subregion. Green: 20–30 Gy subregion. Blue: 10–20 Gy subregion. Cyan: 5–10 Gy subregion.

In this study, seven ROIs were planned for the pretreatment of CT images via Pinnacle3: the PTV, normal lungs, and five ROIs divided by the IDLSS method (5–10 Gy, 10–20 Gy, 20–30 Gy, 30–40 Gy, 40–50 Gy). The PTV was marked by professional radiation oncologists or physicians. Normal lungs were obtained by subtracting the PTV from both lungs, and the IDLSS was derived by subtracting different dose range curves (V_xGy_) of normal lungs from each other, as shown in Figure 2. IDLSS-derived subregions capture dose heterogeneity gradients, enhancing dosiomics feature extraction. These ROIs are used for extracting DVH, radiomics, and dosiomics, as shown in Table 2.

In this study, a preliminary extraction yielded 15 DVH features, 8379 radiomics features, and 735 dosiomics features. The results of feature extraction provide the quantitative data needed for subsequent feature selection and modeling steps to develop the predictive model.

### 3.5. Feature Selection

To remove many redundant features, ANOVA tests were conducted on the radiomic and dosiomic features, and those with a *p* value < 0.05 were retained. The remaining features, along with the clinical and DVH features, were used to create nine different feature combinations for subsequent model development. These combinations aimed to explore the differences in predictive performance between dosiomics and DVH. The feature combinations used in our study are as follows: clinical + DVH/dosiomics (F_DVH,C_, F_D,C_), radiomics + DVH/dosiomics (F_DVH,R_, F_D,R_), clinical + radiomics + DVH/dosiomics (F_DVH,C,R_, F_D,C,R_), and all-features (F_DVH,D,C,R_). Additionally, we included models that exclusively use DVH features (F_DVH_) and those that exclusively use dosiomics features (F_D_). These configurations allow us to rigorously test the predictive capabilities of each set of features both independently and in various combinations, providing a comprehensive analysis of their individual and collective impacts with recent prediction in RP VMAT.

To filter out features related to RP from each feature combination, the least absolute shrinkage and selection operator (LASSO) method was adopted. LASSO employs L1 regularization to impose penalties on model parameters, aiming to reduce the weights of less important features to zero, decreasing model complexity and improving predictive accuracy. This method is effective in handling both continuous and categorical variables and can address issues arising from multicollinearity among independent variables. Moreover, LASSO has the advantage of directly displaying the relationship between features and the prediction target through the positive or negative values of regression coefficients [21].

### 3.6. Model Selection and Evaluation

This study developed and evaluated the LR, RF, XGB and FNN models to compare their predictive performance.

The dataset was split into an 80% training set and a 20% test set via stratified sampling to preserve the ratio of RP-positive and RP-negative cases. The test set was kept completely independent throughout the entire process of model selection and hyperparameter tuning and was only used for the final performance evaluation.

During training, 10-fold cross-validation was performed, where each fold was sequentially used as the validation set, while the remaining folds served as the training set. Within this cross-validation framework, to prevent data leakage, SMOTE was applied only to the training folds, which includes synthetic samples generated by interpolating between existing minority class instances. This cross-validation procedure enabled the evaluation of different parameter combinations, selecting the best-performing configuration for the final model.

Hyperparameter tuning was conducted via a randomized search combined with cross-validation for the LR and RF algorithms. For the FNN, given the larger number of parameters, we additionally applied learning rate scheduling and early stopping to enhance training stability and reduce overfitting.

### 3.7. Performance Evaluation

Following hyperparameter tuning, the final model was trained on the complete training set and evaluated on the independent test set. Model performance was assessed via multiple metrics, including accuracy (ACC), recall (sensitivity), specificity, negative predictive value (PPV), F1 score, and area under the ROC curve (AUC), providing a comprehensive evaluation of the model’s predictive ability.

### 3.8. Decision Curve Analysis (DCA)

To assess the clinical application value of the model under different decision thresholds, this study used DCA to compare the net benefit of each model. DCA was used to evaluate the effectiveness of the model in actual decision scenarios by calculating the trade-off between the gain of true positives and the cost of false positives under different thresholds.

In the DCA, the X-axis represents the decision threshold (threshold probability), and the Y-axis represents the net gain. The net benefit is calculated via Equation (1):

(1) $$Net\;benefit=TP-FP\times \frac{p_{t}}{\;\;1-p_{t\;\;}}\times \frac{1}{N}$$

where TP and FP represent the number of true positives and false positives, respectively, *N* is the total number of samples, and *Pt* is the threshold probability.

The DCA results are compared to those of the “Treat None” and “Treat All” strategies; when the net gain of a model is greater than those of these two strategies at most thresholds, the DCA results are compared with those of the “Treat All” strategies. When the extreme strategy is used, the model has greater decision value in clinical application.

## 4. Results

### 4.1. Impact of the SMOTE on the Class Distribution and Model Performance

Table 3 summarizes the class distributions before and after applying SMOTE to the training set. Prior to balancing, the training set contained 31 RP-positive cases and 77 RP-negative cases, resulting in a positive-to-negative ratio of approximately 1:2.5 After applying the SMOTE, the RP-positive cases were synthetically increased to match the RP-negative cases, achieving a balanced ratio of 1:1.

Using the logistic regression (LR) model to validate the effectiveness of SMOTE, Table 4 systematically presents the performance comparison before and after applying SMOTE. Compared with the original imbalanced dataset, SMOTE markedly increased sensitivity (recall) from 0.42 to 0.68, indicating a substantial improvement in the model’s ability to identify high-risk (RP-positive) patients. Although specificity decreased from 0.88 to 0.71, it remained within an acceptable range, without a pronounced loss in the ability to correctly classify the majority class.

In addition, overall classification performance improved, with the area under the curve (AUC) increasing from 0.71 to 0.76, while the F1-score, which balances precision and recall, increased from 0.41 to 0.56. Overall, models trained on SMOTE-balanced data effectively enhanced the identification of high-risk RP patients without significantly compromising specificity.

### 4.2. Feature Importance Analysis

Table 5 presents a statistical summary of the extracted DVH features related to volume and mean dose. The table displays the DVH characteristics of 136 patients, 97 of whom did not develop RP and 39 of whom did.

For the high-dimensional radiomic and dosiomic feature set, ANOVA was first applied to eliminate redundant features, after which 428 radiomic features and eight dosiomic features were retained for further analysis. Table 6 shows the findings from LASSO regression analysis, which were used to assess the predictive utility of various features for RP in patients who underwent VMAT. This analysis explored nine feature sets and separately reports the number of features before and after ANOVA and LASSO selection, as well as the number of selected features and their importance rankings within each feature set, as detailed in Supplementary Tables S3 and S4.

Radiomics features emerged as highly predictive, consistently securing top rankings across the different sets, thus underscoring their crucial role in accurately predicting RP. The F_D,C_ feature set, with BMI being the sole clinical predictor, also proved significant, demonstrating the effectiveness of a comprehensive approach that integrates diverse types of data to increase the overall predictive accuracy for RP risk. Additionally, DVH features, such as volumes within the 5–10 Gy and 10–20 Gy dose ranges, were included in the analysis.

**Figure 3:**
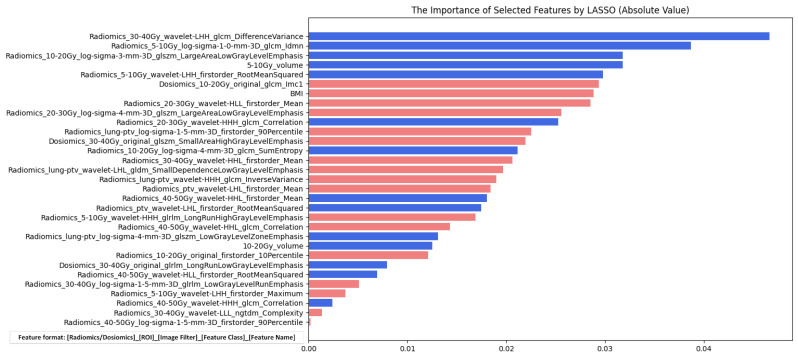
Importance Ranking of Features in the “All-Features” Combination during LASSO Regression Analysis, represented by the Magnitude of Regression Coefficients, with Blue Lines Indicating Negative Correlations and Red Lines Indicating Positive Correlations. Note: Feature names follow the format [ROI][Image Filter][Feature Class]_[Feature Name]. ROI denotes the dose range (e.g., 5–10 Gy, 30–40 Gy) or anatomical region (e.g., lung-GTV, PTV). Image filters include original (no filtering), wavelet-[XYZ] (wavelet decomposition with high/low-frequency components along X, Y, Z directions), and log-sigma-X-mm-3D (Laplacian of Gaussian filtering with sigma X mm). Feature families include first-order, GLCM, GLSZM, GLRLM, GLDM, and NGTDM. (Red Bars (Positive Correlation): These represent features that are positively correlated with the risk of RP. An increase in the value of these features (e.g., higher BMI or specific texture complexities) is associated with a higher probability of the patient developing Grade ≥ 2 RP. Blue Bars (Negative Correlation): These represent features that are negatively correlated with the risk of RP. Higher values in these features (e.g., specific IDLSS subregion volumes like 5–10 Gy) are associated with a lower probability of developing significant RP within this specific model context.)

Figure 3 displays a bar chart illustrating the features selected and their importance determined by LASSO regression analysis from the ‘All-features’ subset (the combination of clinical, DVH, radiomic, and dosiomic features). The length of each bar represents the absolute value of the feature’s importance in the model, with colors indicating the type of feature; red bars represent features positively correlated with the risk of RP, and blue bars represent negatively correlated features. The chart clearly shows that as the bars increase in length, the importance of the feature also increases, reflecting the critical role of texture, shape, or intensity features within different dosage intervals for predicting the risk of RP. The significantly longer bars on the right side highlight the features with the greatest predictive importance for the occurrence of RP.

### 4.3. Model Evaluation

Table 7, Table 8, Table 9 and Table 10 present the predictive performance of the LR, RF, XGB and FNN methods, respectively, under different feature combinations. The results show that, regardless of which model is used, as long as the radiomic feature is included, the predictive performance can be significantly improved. On the other hand, feature combinations without radiomics features (F_DVH_, F_D_, F_C_, and F_D,C_) showed overall inferior performance.

Among all the models, the F_DVH_ combination exhibited the weakest performance, with a significantly lower AUC, F1 score, and sensitivity than the feature sets that included radiomics. The addition of radiomic features consistently increased the predictive performance across all the models. For the RF and FNN, the F_DVH,D,C,R_ combination achieved the highest performance, whereas for the LR and XGB, the F_D,R_ combination demonstrated the best predictive capability.

**Figure 4:**
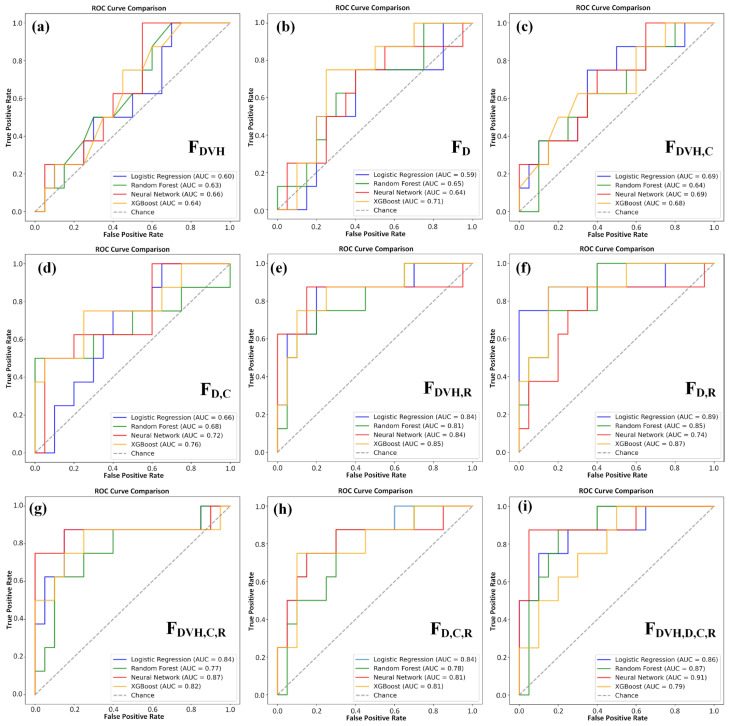
ROC curves for Logistic Regression (LR), Random Forest (RF), Extreme Gradient Boosting (XGB), and Feedforward Neural Network (FNN) models across all feature combinations. (**a**) FDVH, (**b**) FD, (**c**) FDVH,C, (**d**) FD,C, (**e**) FDVH,R (**f**) FD,R, (**g**) FDVH,C,R, (**h**) FD,C,R, (**i**) FDVH,D,C,R. Abbreviations: ROC: Receiver Operating Characteristic, C: Clinical, DVH: Dose–volume Histogram, R: Radiomics, D: Dosiomics, AUC: Area Under the ROC Curve, XGB: Extreme Gradient Boosting.

Figure 4a–i present the ROC curves, which illustrate the performance of each model on both the training and testing datasets. This visualization allows us to assess not only the accuracy of each model but also its ability to generalize beyond the data on which it was trained. The inclusion of ROC curves for both sets ensures a thorough evaluation of model performance and robustness, addressing both overfitting potential and real-world applicability. Their corresponding confusion matrices are provided in Supplementary Figure S1, which facilitates a more intuitive interpretation of classification outcomes by explicitly illustrating true positive, true negative, false positive, and false negative predictions.

**Figure 5:**
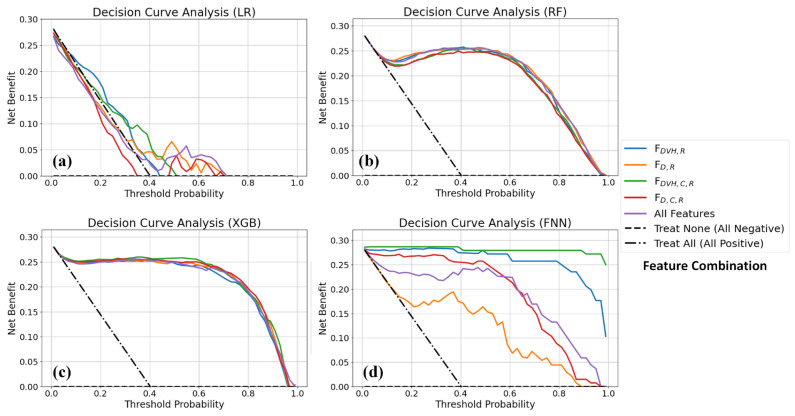
DCA curves for each feature combination in (**a**) LR, (**b**) RF, (**c**) XGB and (**d**) FNN models. Abbreviations: DCA: Decision Curve Analysis, LR: Logistic Regression, RF: Random Forest, XGB: Extreme Gradient Boosting, FNN: Feedforward Neural Network.

Figure 5a–d show the results of the DCA, evaluating the clinical utility of different feature combinations across the four models. Compared with the Treat None and Treat All strategies, the RF and FNN models consistently demonstrated greater net benefits, indicating stable decision support across a range of clinical thresholds. The XGB model exhibited a comparable net benefit profile, performing well at intermediate thresholds, but showed reduced robustness at higher decision thresholds. In contrast, the LR model performed similarly to RF and FNN at lower thresholds (<0.2) but exhibited greater fluctuations at higher thresholds (>0.3), with some combinations showing net benefits close to those of the Treat None strategy, suggesting potential instability in identifying high-risk patients.

Additionally, combinations that excluded radiomic features showed poor performance in terms of both the AUC and DCA, with a net benefit that was significantly lower than that of the Treat None and Treat All strategies. As a result, DCA focused on feature combinations that included radiomic features, further emphasizing the importance of radiomics in enhancing the clinical utility of predictive models.

**Figure 6:**
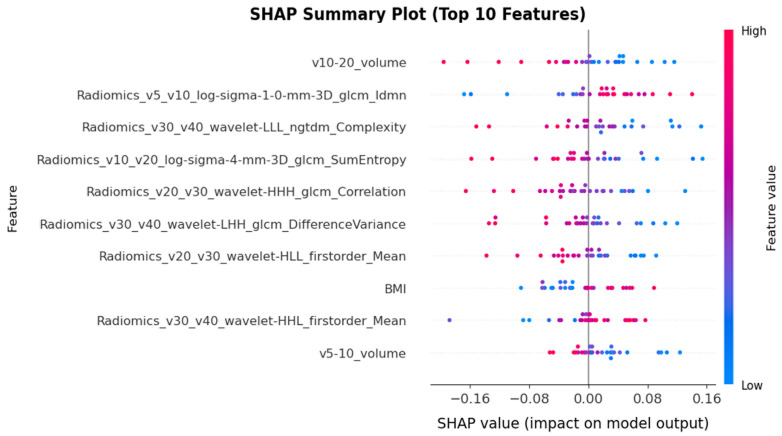
SHAP summary plot of the FNN-based RP prediction model using “All-Features” (DVH, dosimetric, radiomic, and clinical features). Abbreviations: SHAP: SHapley Additive exPlanations, FNN: Feedforward Neural Network, RP: Radiation Pneumonitis, BMI: Body Mass Index, GLCM: Gray-Level Co-occurrence Matrix, NGTDM: Neighborhood Gray-Tone Difference Matrix, LLL/LHH/HHL/HHH: Low–Low–Low/Low–High–High/High–High–Low/High–High–High.

To interpret the predictions of the best-performing FNN model and identify the most influential features, we conducted a SHAP (SHapley Additive exPlanations) analysis. Figure 6 presents the SHAP summary plot of the top 10 features ranked by their SHAP values. The results indicate that volume-based features exhibited the highest predictive importance. Among them, the DVH feature v10–20_volume emerged as the most influential feature, with higher volume values (red dots) predominantly associated with positive SHAP values, indicating an increased likelihood of positive outcomes (RP = 1). This was followed by several radiomic texture features, including Radiomics_v5_v10_log-sigma-1-0-mm-3D_glcm_Idmn and Radiomics_v30_v40_wavelet-LLL_ngtdm_Complexity.

## 5. Discussion

The present findings are consistent with recent advances in pneumonia-related imaging analytics, where machine-learning approaches have been widely applied for pneumonia detection, classification, and clinical decision support [22,23,24]. Emerging evidence indicates that quantitative features extracted from chest radiography and CT can capture subtle parenchymal alterations associated with infectious pneumonia and treatment-related lung injury [23,24]. The field has also evolved from single-image classification toward multimodal integration, ensemble learning, and interpretable modeling frameworks [20,22,24,25,26]. These developments collectively highlight that multimodal machine-learning architectures integrating imaging biomarkers, clinical factors, and radiomics hold greater promise than traditional single-parameter indices for the early identification of patients at elevated risk of lung injury [22].

This study demonstrated a substantial improvement in predicting RP following VMAT by integrating radiomic and dosiomic features extracted from lung subregions via the incremental-dose IDLSS method. Compared with conventional DVH metrics, dosiomics features provided superior predictive performance, highlighting their essential role in capturing spatial dose heterogeneity and enabling more personalized risk assessment. This aligns with previous findings by Chopra et al. [17], who demonstrated the enhanced predictive power of dosiomics for high-grade RP. To further contextualize our findings, a comparative summary of the present study and previously published work is provided in Table 11.

Importantly, the addition of radiomic features further improved the prediction accuracy, particularly in the feature sets F_D,R_ and F_DVH,D,C,R_. This result suggests that radiomics, which captures image texture and intensity information, offers complementary value when combined with dosiomics. The synergistic use of radiomics and dosiomics has been shown to enhance prediction in multiple cancer types and treatment strategies [27,28,29], supporting the value of this combined approach in personalized radiotherapy planning.

However, the predictive power of DVH features alone (the F_DVH_ feature set) remained limited across all the models. This finding is consistent with Bing Li et al. [19], who reported that conventional DVH-based metrics lack sufficient spatial granularity to capture the heterogeneity associated with RP risk, particularly under modern VMAT delivery techniques. These results reinforce the need for advanced spatial dose descriptors, such as dosiomics, to improve prediction accuracy.

### 5.1. Comparison of Model Performance

This study systematically evaluated the predictive capabilities of four machine learning models—LR, RF, XGB and FNN—each offering distinct advantages. LR performs well when lower-dimensional feature sets (e.g., F_D,R_) are used, benefiting from its simplicity, interpretability, and computational efficiency. However, its performance plateaus when handling high-dimensional combinations, reflecting its limited capacity to capture complex, nonlinear relationships.

RF demonstrated robust performance across larger feature sets, particularly F_DVH,D,C,R,_ owing to its ability to model nonlinear feature interactions and manage heterogeneous data types. Nevertheless, RF showed relatively limited performance in identifying the minority class, with a suboptimal sensitivity of 0.50. This limitation may be attributed to the fact that RF primarily optimizes overall node impurity (e.g., the Gini index) during tree splitting, which tends to improve overall classification performance rather than explicitly enhancing recall for the minority class. In imbalanced datasets, this characteristic can bias the model toward the majority class, thereby compromising sensitivity.

Similarly, the XGB model exhibited competitive performance, particularly when radiomics-enriched feature sets were used. The F_D,R_ combination achieved an AUC of 0.87, indicating strong predictive capability. However, performance declined in the high-dimensional all-feature combination (F_DVH,D,C,R_), yielding an AUC of 0.79. This suggests that although XGB effectively captures nonlinear feature interactions through gradient boosting, it may be sensitive to feature redundancy, multicollinearity, and noise inherent in high-dimensional dosiomics spaces. Moreover, decision curve analysis indicated that XGB maintained stable net clinical benefit across intermediate threshold ranges but demonstrated reduced robustness at higher decision thresholds.

In contrast, FNN effectively leveraged its deep learning architecture to uncover complex nonlinear relationships between radiomic and dosiomic features. Although computationally more demanding, FNN offers a clear advantage in learning deep representations from high-dimensional features, making it particularly suitable for modeling dosiomic features that reflect spatial dose heterogeneity. Our findings are consistent with those reported by Wen et al. [30], who showed that a multilayer perceptron (MLP), a specific type of FNN, outperformed RF in RP prediction (MLP: AUC = 0.85, sensitivity = 0.79; RF: AUC = 0.73, sensitivity = 0.57), further supporting the potential advantages of deep learning models for high-dimensional, multimodal data analysis.

### 5.2. Exclusion of SVM and CNN

Models such as SVM and CNN were deliberately excluded after thorough evaluation of their suitability. Although SVM can theoretically handle small-sample, high-dimensional data, its heavy dependence on kernel selection and hyperparameter tuning, along with its lower sensitivity to minority class cases (RP-positive patients), makes it less practical for this imbalanced dataset. The SVM’s emphasis on optimizing overall accuracy rather than sensitivity also limits its clinical utility in this setting.

The CNN, while highly effective in extracting spatial patterns directly from raw images, is less applicable in this study because the features used were preextracted structured features (radiomics and dosiomics) rather than raw CT images. This deliberate exclusion reflects a pragmatic balance between model complexity, interpretability, and compatibility with structured feature vectors, aligning with the study’s objective of developing a clinically applicable and interpretable RP prediction model.

### 5.3. Contribution of Radiomics and Dosiomics to Clinical Decision-Making

The results demonstrate that the RF and FNN models consistently achieved greater net benefits across a wide threshold range, indicating their reliability for clinical decision-making. The XGB model exhibited a comparable net benefit profile, maintaining stable performance across intermediate threshold ranges, which reflects its ability to model nonlinear feature interactions through gradient boosting. Nevertheless, its net benefit declined more rapidly at higher thresholds, suggesting reduced robustness when stricter decision criteria were applied. In contrast, the LR showed greater fluctuations at higher thresholds (≥0.3), suggesting that its linear assumption limits its ability to fully exploit complex feature interactions. Notably, all the high-performing feature combinations included radiomic features, confirming their essential contribution to clinical decision-making.

LASSO regression identified BMI and lung volume within the 5–10 Gy and 10–20 Gy dose ranges as key predictors of RP. These findings align with the work of Rao et al. [31], who demonstrated a positive correlation between higher BMI and increased RP risk in patients receiving radiotherapy. Increased BMI may contribute to setup errors and dose distribution inconsistencies, which, in turn, increase RP risk [31]. This finding reinforces the importance of including BMI in future predictive modeling frameworks for RP risk assessment.

**Table 11:** Comparison of predictive performance (AUC) between the present study and previous studies using different feature types.

Study	DVH-Based AUC	Radiomics + Dosiomics-Based AUC
This study	0.66	0.91
Chopra et al. (2020) [17]	0.63	0.713
Kraus et al. (2023) [32]	0.43	0.79

Abbreviations: DVH: Dose–volume Histogram, AUC: Area Under the ROC Curve.

### 5.4. Values of IDLSS and Advanced Feature Selection

This study employed the IDLSS method, which was originally proposed by Bing Li et al. [19], to improve the spatial precision of feature extraction. By dividing the lung into discrete dose-specific subregions, IDLSS enables the capture of localized dose heterogeneity—information that conventional whole-lung DVH metrics fail to preserve. For each subregion, both radiomic features (describing image texture and shape) and dosiomics features (quantifying spatial dose heterogeneity) were extracted, providing complementary imaging and dosimetric descriptors for the predictive model.

Compared with whole-lung or one-lung segmentation, IDLSS allows finer dose-level division, enabling dosiomics to capture spatial variations more accurately. Kraus et al. [32] reported improved RP prediction (AUC = 0.79) via radiomics and dosiomics, but their approach did not fully address dose heterogeneity. By integrating IDLSS, the present study achieved an AUC of 0.91 with the FNN model, suggesting improved predictive performance within the context of existing approaches, while acknowledging that direct statistical comparisons across studies were not performed.

This multidimensional, spatially resolved approach proved superior to traditional whole-lung DVH analysis, which collapses dose heterogeneity into simple summary statistics (e.g., V_20_, MLD). By preserving localized dose patterns within clinically meaningful dose intervals, IDLSS enhances the sensitivity of feature-based modeling to detect subtle spatial patterns linked to RP development. This refinement is particularly critical under VMAT delivery, where dose distributions are highly heterogeneous. Consistent with the findings of Bing Li et al. [19], the results of this study demonstrate that IDLSS-defined radiomic and dosiomic features significantly enhance prediction accuracy compared with conventional whole-lung DVH analysis, confirming the clinical value of this advanced segmentation strategy.

### 5.5. Predictors in Radiation Pneumonitis Risk

The SHAP value analysis offers profound insights into the features that most strongly influence the predictions of the Feedforward Neural Network (FNN), revealing a hierarchical structure of feature importance. Among the Dose–Volume Histogram (DVH) features, the v10–20_volume parameter exhibits the strongest predictive capability. A positive correlation is observed between higher volume values within the 10–20 Gy dose range and positive SHAP values, indicating that larger irradiated volumes in this specific dose interval (10–20 Gy) are associated with an increased probability of Radiation Pneumonitis (RP). This observation is consistent with the findings of Shepherd et al. [33], who highlighted that low-dose lung irradiation, such as V5 and V10, serves as a critical predictor of RP in patients undergoing Post-Operative Radiation Therapy (PORT). It also aligns with the results reported by Al Feghali et al. [34], where lung density changes were significantly higher in patients experiencing Grade 1 or 2 RP compared to those without RP, particularly within the 10–15 Gy and 15–20 Gy dose bins.

Furthermore, multiple radiomics features derived from various wavelet decompositions and filtering methods rank among the primary predictors. GLCM-based features capture local texture heterogeneity, while wavelet features extract multi-scale information. The emergence of features from different wavelet sub-bands (LLL, HHH, LHH, HHL) suggests that both coarse- and fine-scale texture patterns contribute to recurrence prediction. This agrees with Hirose et al. [35], in whose model for predicting Radiation Pneumonitis (RP), the “Correlation” feature (based on GLCM) was repeatedly selected as a key imaging signature; employing GLCM features in conjunction with wavelet decomposition (multi-scale analysis) effectively captures lung image characteristics and facilitates RP prediction.

### 5.6. Handling High-Dimensional Features

The performance differences observed between LR, RF, and FNN reflect the distinct characteristics and dimensionality of the radiomic and dosiomic features. Radiomics features derived from pretreatment CT images capture primarily texture, intensity, and shape information and are effectively reduced to lower-dimensional sets through LASSO selection. In contrast, dosiomics features inherently possess higher dimensionality owing to their spatial heterogeneity across dose intervals, requiring models with greater capacity to capture complex, nonlinear patterns.

LR, which is suitable for lower-dimensional datasets, performs well in simpler combinations, particularly after LASSO regularization. However, RFs and FNNs are better equipped to handle high-dimensional data. RF’s ability to capture nonlinear feature interactions supported its robust performance with larger feature sets, whereas FNN’s deep learning capacity allowed it to learn multilevel feature representations, further enhancing its predictive accuracy when radiomics and dosiomics features were combined.

Future research could explore dimensionality reduction techniques, such as autoencoders or principal component analysis (PCA), to improve computational efficiency and stability when incorporating high-dimensional features. This would allow for more efficient modeling without sacrificing predictive accuracy.

### 5.7. Potential Clinical Workflow

To bridge the gap between model development and clinical implementation, this study proposes a standardized workflow illustrating how the IDLSS-based prediction framework can be incorporated into radiotherapy clinical decision-making.

The proposed workflow consists of the following four steps:

1. Multimodal Data Integration: During treatment planning, patient clinical parameters (e.g., BMI), pretreatment CT images, and preliminary VMAT dose distributions are obtained from the hospital information system (HIS) and the treatment planning system (TPS).
2. IDLSS Processing and Feature Extraction: Using the incremental-dose interval-based lung subregion segmentation (IDLSS) method, the lung is partitioned into dose-specific subregions ranging from 5 to 50 Gy based on dose gradients. Radiomic and dosiomic features are then extracted from each subregion.
3. Risk Probability Prediction: The extracted features are subsequently input into the best-performing feedforward neural network (FNN) model to estimate the probability of developing Grade ≥ 2 radiation pneumonitis (RP).
4. Clinical Decision Support and Plan Re-optimization: Clinicians may interpret the predicted risk in conjunction with net benefit thresholds derived from decision curve analysis (DCA). When the estimated risk exceeds the recommended threshold, treatment plan re-optimization may be considered, particularly by adjusting dose distributions in key subregions identified by LASSO (e.g., 5–10 Gy or 10–20 Gy intervals) to mitigate RP risk. In addition, patients identified as high-risk may undergo intensified and personalized post-treatment follow-up to facilitate early intervention.

### 5.8. Future Work

Despite the promising performance of our IDLSS-based framework, several limitations must be acknowledged. First, this study was conducted using retrospective data from a single institution. Although internal validation demonstrated high predictive accuracy, the absence of external validation limits the generalizability of our findings to other clinical settings with different treatment protocols or patient demographics.

To address these limitations, future research will focus on three main directions. First, in the absence of immediate access to multicenter data, we will apply transfer learning and fine-tuning strategies using public datasets and external heterogeneous cases to enhance the model’s adaptability across diverse clinical environments.

Second, we plan to establish collaborative partnerships with multiple institutions to construct a diverse, representative database for prospective external validation. This approach enhances the robustness and broader applicability of the model.

Third, dosiomics features in this study were extracted by treating radiation dose values as image grayscale intensities. As a result, these features may be sensitive to dose calculation algorithms and dose grid resolutions employed by different treatment planning systems. Variations in spatial dose distribution gradients introduced by distinct calculation models could affect the stability and reproducibility of dosiomic descriptors. Future multicenter investigations will therefore be essential to evaluate the robustness of dosiomics features across various radiotherapy delivery platforms and dose calculation methods.

Finally, we explore the use of public datasets for preliminary external validation, providing independent confirmation of model performance. Ultimately, we aim to develop a multicenter RP prediction platform that integrates AI-powered clinical decision support tools, facilitating more personalized and accurate radiotherapy planning.

## 6. Conclusions

This study demonstrated that integrating radiomics and dosiomics features extracted from IDLSS-defined lung subregions significantly enhances RP risk prediction after VMAT. Compared with conventional DVH metrics, dosiomics features provide superior predictive accuracy, emphasizing their critical role in capturing spatial dose heterogeneity. Radiomics features further complemented dosiomics by providing image texture information, improving the model’s capacity to detect high-risk patients.

Among the evaluated models, the FNN achieved the highest predictive performance, particularly when radiomics, dosiomics, DVH, and clinical features were combined. Logistic regression (LR) performs well on lower-dimensional combinations but shows limited improvement when handling high-dimensional features, whereas RF provides stable performance across varying feature combinations.

This study highlights the importance of aligning feature selection strategies with suitable modeling approaches. It also emphasizes the value of advanced spatial dose characterization techniques, such as IDLSS, to enhance RP risk prediction. Moving forward, external validation through multicenter collaboration, along with the application of transfer learning, will further enhance the generalizability and clinical utility of this predictive model, contributing to personalized radiation oncology practices and improved patient outcomes.

## Figures and Tables

**Table 1:** Statistical Summary of Patient Clinical Characteristics.

Characteristics	Total	Without RP	With RP	*p* Value
	*n* = 136	*n* = 97 (71.3%)	*n* = 39 (28.7%)	
Age (years)				0.411
Mean	68.9	68.6	69.9	
Range	43–90	49–89	43–90	
Gender				0.636
Male	99	69 (50.7%)	30 (22.1%)	
Female	37	28 (20.6%)	9 (6.6%)	
BMI (kg/m^2^)				<0.05
Mean	23.32	22.68	24.91	
Range	13.66–34.42	13.66–32.87	16.84–34.42	
Tumor classification				0.383
T1	26	19 (14%)	7 (5.1%)	
T2	43	27 (19.9%)	16 (11.8%)	
T3	27	19 (14%)	8 (5.9%)	
T4	40	32 (23.5%)	8 (5.9%)	
Node classification				0.158
N0	45	32 (23.5%)	13 (9.6%)	
N1	15	9 (7%)	6 (4.4%)	
N2	52	42 (30.9%)	10 (7.4%)	
N3	24	14 (10.3%)	10 (7.4%)	
Metastasis classification				0.08
M0	116	79 (58.1%)	107 (78.7%)	
M1	20	18 (13.2%)	2(1.5%)	
Chemotherapy				0.468
Yes	121	88 (64.7%)	33 (24.3%)	
No	15	9 (6.6%)	6 (4.4%)	

Abbreviations: RP: Radiation Pneumonitis, BMI: Body Mass Index.

**Table 2:** Description of the dose–volume histogram (DVH), radiomics, and dosiomics features extracted from each region of interest (ROI).

ROI for Feature Extraction	DVH Features	Radiomics Features	Dosiomics Features
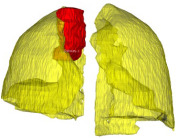 PTV & Normal Lung	PTV: Calculate mean dose and volume Normal Lungs: Calculate the mean dose and V_x_	1197 features for each ROIs Include: Shape, intensity, and texture features extracted from the original images, wavelet filters, and LoG filters.	105 features for each ROIs Include: Shape, intensity, and texture features extracted only from the original images.
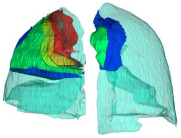 5 Lung subregions (5–10 Gy, 10–20 Gy, 20–30 Gy, 30–40 Gy, and 40–50 Gy)	Calculate the volume of 5 ROIs.		

Abbreviations: DVH: Dose–volume Histogram, PTV: Planning Target Volume, ROI: Region of Interest. Note: The first row summarizes features from the PTV (red) and normal lung (yellow), and the last row summarizes features from the five IDLSS-based lung subregions: 5–10 Gy (cyan), 10–20 Gy (blue), 20–30 Gy (green), 30–40 Gy (yellow), and 40–50 Gy (red). DVH features: Using the open-source platform 3D Slicer, dose–volume histograms for the treatment plan doses and ROIs were calculated, yielding the average dose for the PTV and normal lungs, the volume receiving at least 5 Gy (V_5Gy_), and the corresponding volumes for V_10Gy_, V_20Gy_, V_30Gy_, V_40Gy_, V_50Gy_, and V_60Gy_, as well as the volumes of the ROIs. These features were used to characterize the volumetric distribution of regions of interest across different IDLSS dose intervals and were subsequently included as input variables for statistical analyses and model development. Radiomics features: Using the open-source library PyRadiomics (v3.1.0) in Python (v3.9), features were primarily from 3D pretreatment CT images for each region of interest (ROI) to capture volumetric spatial heterogeneity. The CT images were first processed with wavelet and Laplacian of Gaussian (LoG) filters to detect image details at different scales and orientations. A limited subset of 2D shape features (e.g., maximum 2D diameters) was also included as defined in PyRadiomics. A total of 1197 radiomic features were extracted from each ROI. Detailed feature definitions, parameter settings, and feature counts are provided in Supplementary Tables S1 and S2. Dosiomics features: Treating the dose Gy values as image grayscale intensities, dosiomic features were extracted primarily from 3D dose grids, with selected 2D shape descriptors included, using the same feature definitions and extraction tools as radiomics and without additional filtering. A total of 105 dosiomics features were extracted from each ROI.

**Table 3:** Class distributions before and after applying SMOTE to the training set.

Training Set	RP Positive (Grade ≥ 2)	RP Negative	Ratio (Positive:Negative)
Before SMOTE (Original Data)	31	77	1:2.5
After SMOTE (Training Set Only)	77	77	1:1

Abbreviations: RP: Radiation pneumonitis, SMOTE: Synthetic minority oversampling technique.

**Table 4:** Model performance comparison before and after applying SMOTE (a logistic regression example).

Metric	Before SMOTE	After SMOTE
Sensitivity (Recall)	0.42	0.68
Specificity	0.88	0.71
F1-score	0.41	0.56
AUC	0.71	0.76

Abbreviations: SMOTE: synthetic minority oversampling technique; AUC: area under the ROC curve.

**Table 5:** Statistical Summary of Patient DVH Characteristics, Including Volume and Mean Dose.

DVH Feature	Total (*n* = 136)	Without RP (*n* = 97)	With RP (*n* = 39)
	Range	Range	Range
	Mean ± SD	Mean ± SD	Mean ± SD
PTV volume (cm ^3^)	23.1–1375.9	23.1–1375.9	68.3–818.9
	343.6 ± 240.1	357.5 ± 261.3	309.1 ± 174.8
IDLSS 5–10Gy volume (cm^3^)	44.8–955.2	44.8–955.2	61.3–555.2
	376.7 ± 193.4	394.6 ± 208.6	332.2 ± 142.0
IDLSS 10–20Gy volume (cm^3^)	27.6–904.3	28–904.3	27.6–616.9
	289.9 ± 198.6	298.6 ± 213.9	268.2 ± 154.6
IDLSS 20–30Gy volume (cm^3^)	15.1–354.8	15.1–354.8	18.5–297.9
	114.0 ± 63.4	115.6 ± 65.8	109.9 ± 57.5
IDLSS 30–40Gy volume (cm^3^)	11.7–189.6	11.7–182.7	16–189.9
	79.0 ± 38.4	80.2 ± 38.9	75.9 ± 37.6
IDLSS 40–50Gy volume (cm^3^)	11–194.9	11–180.1	20.2–194.9
	81.5 ± 39.9	80.9 ± 38.7	82.9 ± 43.0
PTV mean dose (Gy)	49.5–74.2	49.5–74.2	50.8–68.8
	61.7 ± 5.1	61.9 ± 5.1	61.3 ± 5.1
Normal lung mean dose (Gy)	1.7–20.7	1.7–20.7	4.9–17.9
	11.1 ± 3.4	11.1 ± 3.6	11.3 ± 2.7

Abbreviations: RP: Radiation Pneumonitis, PTV: Planning Target Volume, IDLSS: Incremental-Dose-interval-based Lung Subregion Segmentation, DVH: Dose–volume Histogram.

**Table 6:** Number of Features Selected and Their Importance Ranking After LASSO Regression Analysis of 7 Feature Combinations.

Feature Sets	Selected Feature Count	Top-Ranked Features
F_DVH_	DVH: 1	5–10Gy_volume
F_D_	Dosiomics: 5	Dosiomics_30–40Gy_original_glszm_SALGLE
		Dosiomics_10–20Gy_original_glcm_Imc1
		Dosiomics_30–40Gy_original_glrlm_LRLGLE
F_DVH,C_	DVH: 1 Clinical: 1	BMI 5–10Gy_volume
F_D,C_	Dosiomics: 4 Clinical: 1	BMI
		Dosiomics_30–40Gy_original_glszm_SALGLE
		Dosiomics_30–40Gy_original_glrlm_LRLGLE
F_DVH,R_	DVH: 2 Radiomics: 30	Radiomics_30–40Gy_wavelet-LHH_glcm_DifferenceVariance
		Radiomics_10–20Gy_log-sigma-3-mm-3D_glszm_LALGLE
		Radiomics_20–30Gy_log-sigma-4-mm-3D_glszm_LALGLE
F_D,R_	Dosiomics: 3 Radiomics: 30	Radiomics_30–40Gy_wavelet-LHH_glcm_DifferenceVariance
		Radiomics_20–30Gy_wavelet-HLL_firstorder_Mean
		Radiomics_5–10Gy_log-sigma-1-0-mm-3D_glcm_Idmn
F_DVH,C,R_	DVH: 2 Clinical: 1 Radiomics: 27	Radiomics_30–40Gy_wavelet-LHH_glcm_DifferenceVariance
		Radiomics_10–20Gy_log-sigma-3-mm-3D_glszm_LALGLE
		Radiomics_5–10Gy_log-sigma-1-0-mm-3D_glcm_Idmn
F_D,C,R_	Dosiomics: 3 Clinical: 1 Radiomics: 31	Radiomics_30–40Gy_wavelet-LHH_glcm_DifferenceVariance
		Radiomics_20–30Gy_wavelet-HLL_firstorder_Mean
		Dosiomics_10–20Gy_original_glcm_Imc1
F_DVH,D,C,R_	DVH: 2 Dosiomics: 3 Clinical: 1 Radiomics: 25	Radiomics_30–40Gy_wavelet-LHH_glcm_DifferenceVariance
		Radiomics_5–10Gy_log-sigma-1-0-mm-3D_glcm_Idmn
		Radiomics_10–20Gy_log-sigma-3-mm-3D_glszm_LALGLE

Abbreviations: DVH: Dose–volume Histogram, GLCM: Gray Level Co-occurrence Matrix, GLSZM: Gray Level Size Zone Matrix, GLRLM: Gray Level Run Length Matrix, LoG: Laplacian of Gaussian, BMI: Body Mass Index. Please note: This table displays the number of features retained after LASSO regression analysis and the top-ranked features, up to a maximum of three, within each feature subset.

**Table 7:** Performance metrics of Logistic Regression models evaluated on the independent test set with nine types of feature combinations.

Features Sets	AUC	ACC	NPV	PPV	F1-Score	Sensitivity	Specificity
F_DVH_	0.60	0.46	0.73	0.29	0.40	0.63	0.40
F_D_	0.59	0.64	0.73	0.33	0.29	0.25	0.80
F_DVH,C_	0.69	0.61	0.77	0.36	0.42	0.50	0.65
F_D,C_	0.66	0.61	0.77	0.36	0.42	0.50	0.65
F_DVH,R_	0.84	0.79	0.94	0.58	0.70	0.88	0.75
F_D,R_	0.89	0.79	0.94	0.58	0.70	0.88	0.75
F_DVH,C,R_	0.84	0.79	0.94	0.58	0.70	0.88	0.75
F_D,C,R_	0.84	0.68	0.92	0.47	0.61	0.88	0.60
F_DVH,D,C,R_	0.86	0.79	0.94	0.58	0.70	0.88	0.75

Abbreviations: C: Clinical, DVH: Dose–volume Histogram, R: Radiomics, D: Dosiomics, AUC: Area Under the ROC Curve, ACC: Accuracy, NPV: Negative Predictive Value, PPV: Positive Predictive Value.

**Table 8:** Performance metrics of Random Forest models evaluated on the independent test set with nine types of feature combinations.

Features Sets	AUC	ACC	NPV	PPV	F1-Score	Sensitivity	Specificity
F_DVH_	0.63	0.64	0.78	0.40	0.44	0.50	0.70
F_D_	0.65	0.68	0.79	0.44	0.47	0.50	0.75
F_DVH,C_	0.64	0.64	0.78	0.40	0.44	0.50	0.70
F_D,C_	0.68	0.64	0.81	0.42	0.50	0.63	0.65
F_DVH,R_	0.81	0.79	0.79	0.75	0.50	0.38	0.95
F_D,R_	0.85	0.79	0.79	0.75	0.50	0.38	0.95
F_DVH,C,R_	0.77	0.79	0.82	0.67	0.57	0.50	0.90
F_D,C,R_	0.78	0.75	0.78	0.60	0.46	0.38	0.90
F_DVH,D,C,R_	0.87	0.79	0.82	0.67	0.57	0.50	0.90

Abbreviations: C: Clinical, DVH: Dose–volume Histogram, R: Radiomics, D: Dosiomics, AUC: Area Under the ROC Curve, ACC: Accuracy, NPV: Negative Predictive Value, PPV: Positive Predictive Value.

**Table 9:** Performance metrics of Extreme Gradient Boosting models evaluated on the independent test set with nine types of feature combinations.

Features Sets	AUC	ACC	NPV	PPV	F1-Score	Sensitivity	Specificity
F_DVH_	0.64	0.64	0.73	0.33	0.29	0.25	0.80
F_D_	0.71	0.68	0.76	0.43	0.40	0.38	0.80
F_DVH,C_	0.68	0.64	0.81	0.42	0.50	0.63	0.65
F_D,C_	0.76	0.68	0.79	0.44	0.47	0.50	0.75
F_DVH,R_	0.85	0.79	0.82	0.67	0.57	0.50	0.90
F_D,R_	0.87	0.79	0.79	0.75	0.50	0.38	0.95
F_DVH,C,R_	0.82	0.82	0.86	0.71	0.67	0.63	0.90
F_D,C,R_	0.81	0.79	0.82	0.67	0.57	0.50	0.90
F_DVH,D,C,R_	0.79	0.75	0.78	0.60	0.46	0.38	0.90

Abbreviations: C: Clinical, DVH: Dose–volume Histogram, R: Radiomics, D: Dosiomics, AUC: Area Under the ROC Curve, ACC: Accuracy, NPV: Negative Predictive Value, PPV: Positive Predictive Value.

**Table 10:** Performance metrics of Feedforward Neural Network models evaluated on the independent test set with nine types of feature combinations.

Features Sets	AUC	ACC	NPV	PPV	F1-Score	Sensitivity	Specificity
F_DVH_	0.66	0.54	1.00	0.38	0.55	1.00	0.35
F_D_	0.64	0.57	0.83	0.38	0.50	0.75	0.50
F_DVH,C_	0.69	0.54	0.82	0.35	0.48	0.75	0.45
F_D,C_	0.73	0.75	0.81	0.57	0.53	0.50	0.85
F_DVH,R_	0.84	0.86	0.94	0.70	0.78	0.88	0.85
F_D,R_	0.74	0.71	0.80	0.50	0.50	0.50	0.80
F_DVH,C,R_	0.87	0.82	0.90	0.67	0.71	0.75	0.85
F_D,C,R_	0.81	0.79	0.89	0.60	0.67	0.75	0.80
F_DVH,D,C,R_	0.91	0.89	0.95	0.78	0.82	0.88	0.90

Abbreviations: C: Clinical, DVH: Dose–volume Histogram, R: Radiomics, D: Dosiomics, AUC: Area Under the ROC Curve, ACC: Accuracy, NPV: Negative Predictive Value, PPV: Positive Predictive Value.

## Data Availability

The datasets generated and/or analyzed during the current study are not publicly available owing to legal and ethical restrictions but are available from the corresponding author upon reasonable request and with appropriate institutional approval.

## Supplementary Materials

The following supporting information can be downloaded at: https://www.mdpi.com/article/10.3390/life16020328/s1, Figure S1: Confusion matrices of LR, RF, XGBoost, and FNN models on the independent test set using the All-Features combination; Table S1: PyRadiomics Feature Extraction Configuration for Dosiomics and Radiomics Analysis; Table S2: Number of radiomic features extracted from each image type and feature class for a single ROI. Table S3: Summary of feature selection results using ANOVA and LASSO across different feature types. Table S4: Detailed Features of the 7 Feature Subsets Identified by LASSO.
