# Supplementary material for: Improving the Prediction of Radiation Pneumonitis: Leveraging Radiomics and Dosiomics Within IDLSS Lung Subregions
Source: Life (Basel). 2026 Feb 13;16(2):328. doi: 10.3390/life16020328 (PMC12941900; doi:10.3390/life16020328)
Supplement: Supplementary file 1 [file life-16-00328-s001.zip › life-4071044-supplementary.pdf]

Supplementary

Supplementary Table S1. PyRadiomics Feature Extraction Configuration for Dosiomics and Radiomics Analysis

	Dosiomics	Radiomics
imageType	Original: {}	Original: {}<br>LoG:<br>sigma: [1.0, 1.5, 3, 4]<br>Wavelet: {}
featureClass	shape:<br>firstorder:<br>glcm:<br>- 'Autocorrelation'<br>- 'JointAverage'<br>- 'ClusterProminence'<br>- 'ClusterShade'<br>- 'ClusterTendency'<br>- 'Contrast'<br>- 'Correlation'<br>- 'DifferenceAverage'<br>- 'DifferenceEntropy'<br>- 'DifferenceVariance'<br>- 'JointEnergy'<br>- 'JointEntropy'<br>- 'Imc1'<br>- 'Imc2'<br>- 'Idm'<br>- 'Idmn'<br>- 'Id'<br>- 'Idn'<br>- 'InverseVariance'<br>- 'MaximumProbability'<br>- 'SumEntropy'<br>- 'SumSquares'<br>glrlm:<br>glszm:<br>gldm:<br>ngtdm:	shape:<br>firstorder:<br>glcm:<br>- 'Autocorrelation'<br>- 'JointAverage'<br>- 'ClusterProminence'<br>- 'ClusterShade'<br>- 'ClusterTendency'<br>- 'Contrast'<br>- 'Correlation'<br>- 'DifferenceAverage'<br>- 'DifferenceEntropy'<br>- 'DifferenceVariance'<br>- 'JointEnergy'<br>- 'JointEntropy'<br>- 'Imc1'<br>- 'Imc2'<br>- 'Idm'<br>- 'Idmn'<br>- 'Id'<br>- 'Idn'<br>- 'InverseVariance'<br>- 'MaximumProbability'<br>- 'SumEntropy'<br>- 'SumSquares'<br>glrlm:<br>glszm:<br>gldm:<br>ngtdm:
binWidth	25	25
voxelArrayShift	1000	1000

Abbreviation: LoG: Laplacian of Gaussian, GLCM: Gray Level Co-occurrence Matrix, GLSZM: Gray Level Size Zone Matrix, GLRLM: Gray Level Run Length Matrix, NGTDM: Neighbouring Gray Tone Difference Matrix, GLDM: Gray Level Dependence Matrix, LoG: Laplacian of Gaussian

Supplementary Table S2. Number of radiomic features extracted from each image type and feature class for a single ROI.

Image type	Filter setting	Shape	First-order	GLCM	GLDM	GLRLM	GLSZM	NGTDM	Total
**Original image**	-	14	18	22	14	16	16	5	105
**LoG-filtered images**	σ = 1.0 mm, 3D	0	18	22	14	16	16	5	91
	σ = 1.5 mm, 3D	0	18	22	14	16	16	5	91
	σ = 3.0 mm, 3D	0	18	22	14	16	16	5	91
	σ = 4.0 mm, 3D	0	18	22	14	16	16	5	91
**Wavelet-decomposed images**	HHH	0	18	22	14	16	16	5	91
	HHL	0	18	22	14	16	16	5	91
	HLH	0	18	22	14	16	16	5	91
	HLL	0	18	22	14	16	16	5	91
	LHH	0	18	22	14	16	16	5	91
	LHL	0	18	22	14	16	16	5	91
	LLH	0	18	22	14	16	16	5	91
	LLL	0	18	22	14	16	16	5	91
**Number of radiomic features per ROI**		14	234	286	182	208	208	65	1197

Abbreviation: LoG: Laplacian of Gaussian, GLCM: Gray Level Co-occurrence Matrix, GLSZM: Gray Level Size Zone Matrix, GLRLM: Gray Level Run Length Matrix, NGTDM: Neighbouring Gray Tone Difference Matrix, GLDM: Gray Level Dependence Matrix, LoG: Laplacian of Gaussian, ROI: Region of Interest

***Note:* H and L denote high-pass and low-pass filtering along each spatial dimension.**

Supplementary Table S3. Summary of feature selection results using ANOVA and LASSO across different feature types.

Feature type	Initial number of features	After ANOVA selection	After LASSO selection
DVH features	15	15 (ANOVA not applied)	2
Clinical features	7	7 (ANOVA not applied)	1
Dosiomics features	735	8	3
Radiomics features	8,379	428	25
Total	9,136	458	31

Abbreviation: DVH: Dose-volume Histogram, ANOVA: Analysis of Variance, LASSO：Least Absolute Shrinkage and Selection Operator

Supplementary Table S4. Detailed Features of the 7 Feature Subsets Identified by LASSO

Features sets	Selected Features Count	All features in subset
$F_{DVH,C}$	DVH: 1<br>Clinical: 1	BMI<br>5-10Gy_volume
$F_{D,C}$	Dosiomics: 4<br>Clinical: 1	Dosiomics_v10_v20_original_firstorder_Maximum<br>Dosiomics_v10_v20_original_glcm_Imc1<br>Dosiomics_v30_v40_original_glrlm_LongRunLowGrayLevelEmphasis<br>Dosiomics_v30_v40_original_glszm_SmallAreaHighGrayLevelEmphasis<br>BMI
$F_{DVH,R}$	DVH: 2<br>Radiomics: 30	v10-20_volume<br>v5-10_volume<br>Radiomics_ptv_wavelet-LHL_firstorder_Mean<br>Radiomics_ptv_wavelet-LHL_firstorder_RootMeanSquared<br>Radiomics_ptv_wavelet-LHL_gldm_DependenceNonUniformityNormalized<br>Radiomics_ normallungs _log-sigma-1-5-mm-3D_firstorder_90Percentile<br>Radiomics_ normallungs _log-sigma-3-mm-3D_firstorder_90Percentile<br>Radiomics_normallungs_log-sigma-4-mm-3D_glszm_LowGrayLevelZoneEmphasis<br>Radiomics_ normallungs _wavelet-LHL_gldm_SmallDependenceLowGrayLevelEmphasis<br>Radiomics_ normallungs _wavelet-LHH_glszm_ZoneEntropy<br>Radiomics_ normallungs _wavelet-HLL_glcm_Idmn<br>Radiomics_ normallungs _wavelet-HHH_glcm_InverseVariance<br>Radiomics_v5_v10_log-sigma-1-0-mm-3D_glcm_Idmn<br>Radiomics_v5_v10_wavelet-LHH_firstorder_Maximum

		Radiomics_v5_v10_wavelet-LHH_firstorder_RootMeanSquared<br>Radiomics_v5_v10_wavelet-HHH_glcm_JointAverage<br>Radiomics_v5_v10_wavelet-HHH_glrlm_LongRunHighGrayLevelEmphasis<br>Radiomics_v10_v20_original_firstorder_10Percentile<br>Radiomics_v10_v20_log-sigma-3-mm-3D_glszm_LargeAreaLowGrayLevelEmphasis<br>Radiomics_v10_v20_log-sigma-4-mm-3D_glcm_SumEntropy<br>Radiomics_v10_v20_log-sigma-4-mm-3D_glszm_LargeAreaLowGrayLevelEmphasis<br>Radiomics_v20_v30_log-sigma-4-mm-3D_glszm_LargeAreaLowGrayLevelEmphasis<br>Radiomics_v20_v30_wavelet-HLL_firstorder_Mean<br>Radiomics_v20_v30_wavelet-HHH_glcm_Correlation<br>Radiomics_v30_v40_log-sigma-1-5-mm-3D_glrlm_LowGrayLevelRunEmphasis<br>Radiomics_v30_v40_wavelet-LHH_glcm_DifferenceVariance<br>Radiomics_v30_v40_wavelet-HHL_firstorder_Mean<br>Radiomics_v40_v50_log-sigma-1-5-mm-3D_firstorder_90Percentile<br>Radiomics_v40_v50_wavelet-HLL_firstorder_RootMeanSquared<br>Radiomics_v40_v50_wavelet-HHL_firstorder_Mean<br>Radiomics_v40_v50_wavelet-HHL_glcm_Correlation<br>Radiomics_v40_v50_wavelet-HHH_glcm_Correlation
$F_{D,R}$	Dosiomics: 3<br>Radiomics: 30	Dosiomics_v10_v20_original_glcm_Imc1<br>Dosiomics_v30_v40_original_glrlm_ShortRunHighGrayLevelEmphasis<br>Dosiomics_v30_v40_original_glszm_SmallAreaHighGrayLevelEmphasis

		Radiomics_ptv_wavelet-LHL_firstorder_Mean<br><br>Radiomics_ptv_wavelet-LHL_firstorder_RootMeanSquared<br><br>Radiomics_ normallungs _log-sigma-1-5-mm-3D_firstorder_90Percentile<br><br>Radiomics_ normallungs _log-sigma-3-mm-3D_firstorder_90Percentile<br><br>Radiomics_ normallungs _log-sigma-4-mm-3D_glszm_LowGrayLevelZoneEmphasis<br><br>Radiomics_ normallungs _wavelet-LHL_gldm_SmallDependenceLowGrayLevelEmphasis<br><br>Radiomics_ normallungs _wavelet-LHH_glszm_ZoneEntropy<br><br>Radiomics_ normallungs _wavelet-HHH_glcm_InverseVariance<br><br>Radiomics_v5_v10_log-sigma-1-0-mm-3D_glcm_Idmn<br><br>Radiomics_v5_v10_wavelet-LLH_glszm_SizeZoneNonUniformity<br><br>Radiomics_v5_v10_wavelet-LHH_firstorder_Maximum<br><br>Radiomics_v5_v10_wavelet-LHH_firstorder_RootMeanSquared<br><br>Radiomics_v5_v10_wavelet-HHH_glrlm_LongRunHighGrayLevelEmphasis<br><br>Radiomics_v5_v10_wavelet-LLL_firstorder_TotalEnergy<br><br>Radiomics_v10_v20_original_firstorder_10Percentile<br><br>Radiomics_v10_v20_log-sigma-3-mm-3D_glszm_LargeAreaLowGrayLevelEmphasis<br><br>Radiomics_v10_v20_log-sigma-4-mm-3D_glcm_SumEntropy<br><br>Radiomics_v20_v30_log-sigma-4-mm-3D_glszm_LargeAreaLowGrayLevelEmphasis<br><br>Radiomics_v20_v30_wavelet-HLL_firstorder_Mean<br><br>Radiomics_v20_v30_wavelet-HHH_glcm_Correlation<br><br>Radiomics_v30_v40_log-sigma-1-5-mm-

		3D_glrlm_LowGrayLevelRunEmphasis<br><br>Radiomics_v30_v40_wavelet-LHL_glcm_Imc2<br><br>Radiomics_v30_v40_wavelet-LHH_glcm_DifferenceVariance<br><br>Radiomics_v30_v40_wavelet-HHL_firstorder_Mean<br><br>Radiomics_v30_v40_wavelet-LLL_ngtdm_Complexity<br><br>Radiomics_v40_v50_log-sigma-1-5-mm-3D_firstorder_90Percentile<br><br>Radiomics_v40_v50_wavelet-HLL_firstorder_RootMeanSquared<br><br>Radiomics_v40_v50_wavelet-HHL_firstorder_Mean<br><br>Radiomics_v40_v50_wavelet-HHL_glcm_Correlation<br><br>Radiomics_v40_v50_wavelet-HHH_glcm_Correlation
$F_{DVH,C,R}$	DVH: 2<br>Clinical: 1<br>Radiomics: 27	v10-20_volume<br><br>v5-10_volume<br><br>BMI<br><br>Radiomics_ normallungs _log-sigma-1-5-mm-3D_firstorder_90Percentile<br><br>Radiomics_ normallungs _log-sigma-4-mm-3D_glszm_LowGrayLevelZoneEmphasis<br><br>Radiomics_ normallungs _wavelet-HHH_glcm_InverseVariance<br><br>Radiomics_ normallungs _wavelet-HLL_glcm_Idmn<br><br>Radiomics_ normallungs _wavelet-LHL_gldm_SmallDependenceLowGrayLevelEmphasis<br><br>Radiomics_ normallungs _wavelet-LHL_glszm_LargeAreaHighGrayLevelEmphasis<br><br>Radiomics_ptv_wavelet-LHL_firstorder_Mean<br><br>Radiomics_ptv_wavelet-LHL_firstorder_RootMeanSquared

		Radiomics_ptv_wavelet-LHL_gldm_DependenceNonUniformityNormalized<br>Radiomics_v10_v20_log-sigma-3-mm-3D_glszm_LargeAreaLowGrayLevelEmphasis<br>Radiomics_v10_v20_log-sigma-4-mm-3D_glcm_SumEntropy<br>Radiomics_v10_v20_log-sigma-4-mm-3D_glszm_LargeAreaLowGrayLevelEmphasis<br>Radiomics_v10_v20_original_firstorder_10Percentile<br>Radiomics_v20_v30_log-sigma-4-mm-3D_glszm_LargeAreaLowGrayLevelEmphasis<br>Radiomics_v20_v30_wavelet-HHH_glcm_Correlation<br>Radiomics_v20_v30_wavelet-HLL_firstorder_Mean<br>Radiomics_v30_v40_log-sigma-1-5-mm-3D_glrlm_LowGrayLevelRunEmphasis<br>Radiomics_v30_v40_wavelet-HHL_firstorder_Mean<br>Radiomics_v30_v40_wavelet-LHH_glcm_DifferenceVariance<br>Radiomics_v40_v50_wavelet-HHH_glcm_Correlation<br>Radiomics_v40_v50_wavelet-HHL_firstorder_Mean<br>Radiomics_v40_v50_wavelet-HHL_glcm_Correlation<br>Radiomics_v40_v50_wavelet-HLL_firstorder_RootMeanSquared<br>Radiomics_v5_v10_log-sigma-1-0-mm-3D_glcm_Idmn<br>Radiomics_v5_v10_wavelet-HHH_glrlm_LongRunHighGrayLevelEmphasis<br>Radiomics_v5_v10_wavelet-LHH_firstorder_Maximum<br>Radiomics_v5_v10_wavelet-LHH_firstorder_RootMeanSquared

$F_{D,C,R}$	Dosiomics: 3<br>Clinical: 1<br>Radiomics: 31	BMI<br>Dosiomics_v10_v20_original_glcm_Imc1<br>Dosiomics_v30_v40_original_glrlm_LongRunLowGrayLevelEmphasis<br>Dosiomics_v30_v40_original_glszm_SmallAreaHighGrayLevelEmphasis<br>Radiomics_ normallungs _log-sigma-1-5-mm-3D_firstorder_90Percentile<br>Radiomics_ normallungs _log-sigma-3-mm-3D_firstorder_90Percentile<br>Radiomics_ normallungs _log-sigma-4-mm-3D_glszm_LowGrayLevelZoneEmphasis<br>Radiomics_ normallungs _wavelet-HHH_glcm_InverseVariance<br>Radiomics_ normallungs _wavelet-LHH_glszm_ZoneEntropy<br>Radiomics_ normallungs _wavelet-LHL_gldm_SmallDependenceLowGrayLevelEmphasis<br>Radiomics_ normallungs _wavelet-LHL_glszm_LargeAreaHighGrayLevelEmphasis<br>Radiomics_ptv_wavelet-LHL_firstorder_Mean<br>Radiomics_ptv_wavelet-LHL_firstorder_RootMeanSquared<br>Radiomics_v10_v20_log-sigma-3-mm-3D_glszm_LargeAreaLowGrayLevelEmphasis<br>Radiomics_v10_v20_log-sigma-4-mm-3D_glcm_SumEntropy<br>Radiomics_v10_v20_original_firstorder_10Percentile<br>Radiomics_v10_v20_wavelet-LHH_glrlm_RunEntropy<br>Radiomics_v20_v30_log-sigma-4-mm-3D_glszm_LargeAreaLowGrayLevelEmphasis<br>Radiomics_v20_v30_wavelet-HHH_glcm_Correlation<br>Radiomics_v20_v30_wavelet-HLL_firstorder_Mean

		Radiomics_v30_v40_log-sigma-1-5-mm-3D_glrlm_LowGrayLevelRunEmphasis<br>Radiomics_v30_v40_wavelet-HHL_firstorder_Mean<br>Radiomics_v30_v40_wavelet-LHH_glcm_DifferenceVariance<br>Radiomics_v30_v40_wavelet-LLL_ngtdm_Complexity<br>Radiomics_v40_v50_log-sigma-1-5-mm-3D_firstorder_90Percentile<br>Radiomics_v40_v50_wavelet-HHH_glcm_Correlation<br>Radiomics_v40_v50_wavelet-HHL_firstorder_Mean<br>Radiomics_v40_v50_wavelet-HHL_glcm_Correlation<br>Radiomics_v40_v50_wavelet-HLL_firstorder_RootMeanSquared<br>Radiomics_v5_v10_log-sigma-1-0-mm-3D_glcm_Idmn<br>Radiomics_v5_v10_wavelet-HHH_glrlm_LongRunHighGrayLevelEmphasis<br>Radiomics_v5_v10_wavelet-LHH_firstorder_Maximum<br>Radiomics_v5_v10_wavelet-LHH_firstorder_RootMeanSquared<br>Radiomics_v5_v10_wavelet-LLH_glszm_SizeZoneNonUniformity<br>Radiomics_v5_v10_wavelet-LLL_firstorder_TotalEnergy
All features	DVH: 2<br>Dosiomics: 3<br>Clinical: 1<br>Radiomics: 25	v10-20_volume<br>v5-10_volume<br>Dosiomics_v10_v20_original_glcm_Imc1<br>Dosiomics_v30_v40_original_glrlm_LongRunLowGrayLevelEmphasis<br>Dosiomics_v30_v40_original_glszm_SmallAreaHighGrayLevelEmphasis<br>BMI<br>Radiomics_ normallungs _log-sigma-1-5-mm-3D_firstorder_90Percentile<br>Radiomics_ normallungs _log-sigma-4-mm-

		3D_glszm_LowGrayLevelZoneEmphasis<br><br>Radiomics_ normallungs _wavelet-HHH_glcm_InverseVariance<br><br>Radiomics_ normallungs _wavelet-<br>LHL_gldm_SmallDependenceLowGrayLevelEmphasis<br><br>Radiomics_ptv_wavelet-LHL_firstorder_Mean<br><br>Radiomics_ptv_wavelet-LHL_firstorder_RootMeanSquared<br><br>Radiomics_v10_v20_log-sigma-3-mm-<br>3D_glszm_LargeAreaLowGrayLevelEmphasis<br><br>Radiomics_v10_v20_log-sigma-4-mm-3D_glcm_SumEntropy<br><br>Radiomics_v10_v20_original_firstorder_10Percentile<br><br>Radiomics_v20_v30_log-sigma-4-mm-<br>3D_glszm_LargeAreaLowGrayLevelEmphasis<br><br>Radiomics_v20_v30_wavelet-HHH_glcm_Correlation<br><br>Radiomics_v20_v30_wavelet-HLL_firstorder_Mean<br><br>Radiomics_v30_v40_log-sigma-1-5-mm-<br>3D_glrlm_LowGrayLevelRunEmphasis<br><br>Radiomics_v30_v40_wavelet-HHL_firstorder_Mean<br><br>Radiomics_v30_v40_wavelet-LHH_glcm_DifferenceVariance<br><br>Radiomics_v30_v40_wavelet-LLL_ngtdm_Complexity<br><br>Radiomics_v40_v50_log-sigma-1-5-mm-3D_firstorder_90Percentile<br><br>Radiomics_v40_v50_wavelet-HHH_glcm_Correlation<br><br>Radiomics_v40_v50_wavelet-HHL_firstorder_Mean<br><br>Radiomics_v40_v50_wavelet-HHL_glcm_Correlation<br><br>Radiomics_v40_v50_wavelet-HLL_firstorder_RootMeanSquared<br><br>Radiomics_v5_v10_log-sigma-1-0-mm-3D_glcm_Idmn

		Radiomics_v5_v10_wavelet-HHH_glrlm_LongRunHighGrayLevelEmphasis<br>Radiomics_v5_v10_wavelet-LHH_firstorder_Maximum<br>Radiomics_v5_v10_wavelet-LHH_firstorder_RootMeanSquared

Abbreviation: DVH: Dose-volume Histogram, GLCM: Gray Level Co-occurrence Matrix, GLSZM: Gray Level Size Zone Matrix, GLRLM: Gray Level Run Length Matrix, NGTDM: Neighbouring Gray Tone Difference Matrix, GLDM: Gray Level Dependence Matrix, LoG: Laplacian of Gaussian, BMI: Body Mass Index

Supplementary Figures

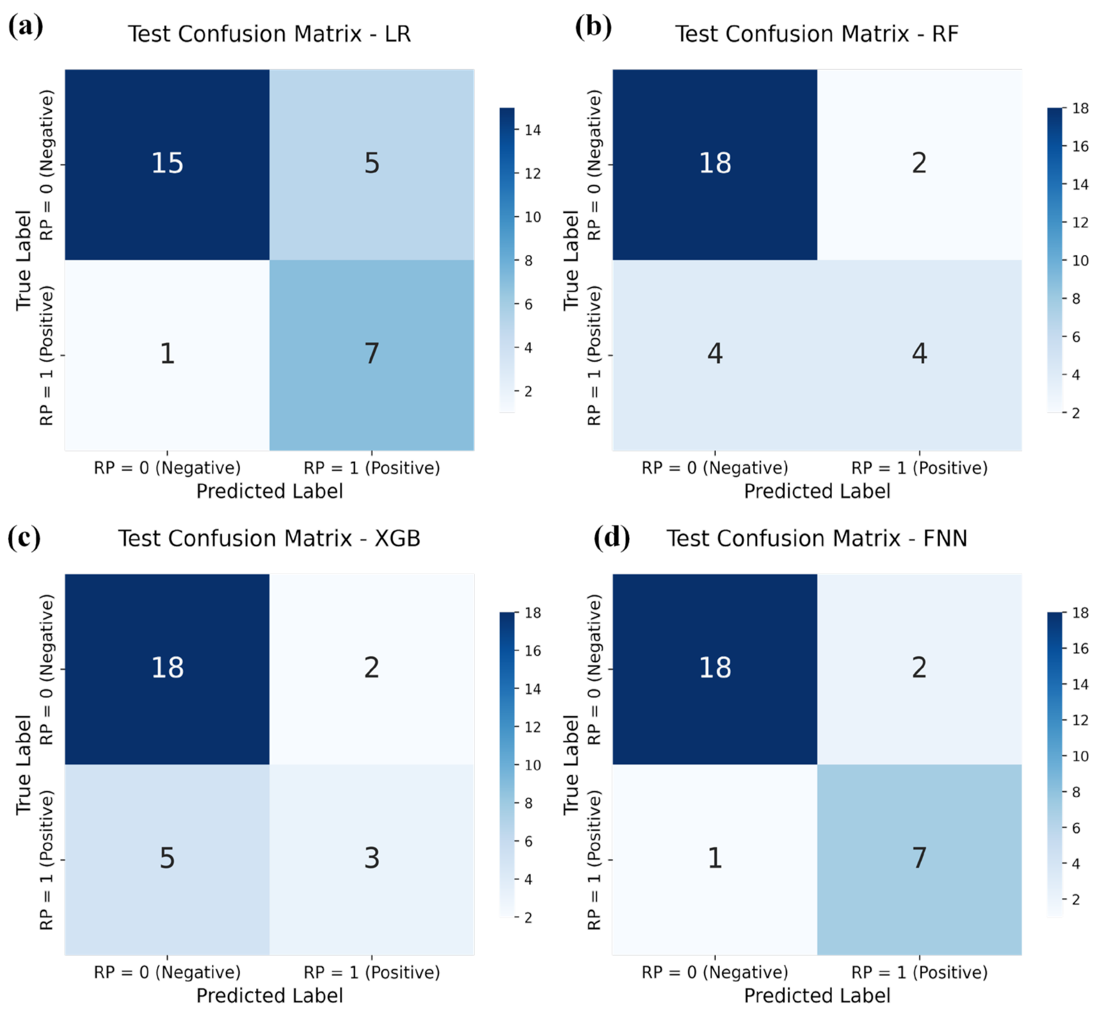

Supplementary Figure S1. Confusion matrices of LR, RF, XGBoost, and FNN models on the independent test set using the All-Features combination.

Abbreviation: Logistic Regression; RF, Random Forest; XGB, Extreme Gradient Boosting (XGBoost); FNN, Feedforward Neural Network; RP, Radiation Pneumonitis; DVH, Dose–Volume Histogram.
